# Supplementary material for: Estimating the association between blood pressure variability and cardiovascular disease: An application using the ARIC Study
Source: Stat Med. 2018 Dec 21;38(10):1855–68. doi: 10.1002/sim.8074 (PMC6445736; doi:10.1002/sim.8074)
Supplement: Supplementary file 1 — SIM_8074‐Supp‐0001‐supp_material.pdf [file SIM-38-1855-s001.pdf]

Supplementary Materials to 'Estimating the association between blood pressure variability and cardiovascular disease: An application using the ARIC Study'

Jessica Barrett, Raphael Huille, Richard Parker, Yuichiro Yano and Michael Griswold

1 Bias caused by ignoring correlations in linear regression

Consider a linear regression of outcome y on correlated covariates x_1 and x_2,

$$y = \beta_1 x_1 + \beta_2 x_2 + \varepsilon \ ,$$

where $\varepsilon \sim N(0, \sigma_y^2)$ is the residual error in y. Now let

$$x_1 = \rho \frac{\sigma_1}{\sigma_2} x_2 + \lambda \ ,$$

where λ and x_2 are uncorrelated, $\mathrm{Var}(x_1) = \sigma_1^2$, $\mathrm{Var}(x_2) = \sigma_2^2$ and ρ is the correlation between x_1 and x_2. Re-writing the linear predictor,

$$y = \beta_1 \lambda + \left(\rho \frac{\sigma_1}{\sigma_2} \beta_1 + \beta_2 \right) x_2 \ .$$

As λ and x_2 are uncorrelated we find

$$\hat{\beta}_2 = \frac{\mathrm{Cov}(y, x_2)}{\sigma_2^2} - \rho \hat{\beta}_1 \frac{\sigma_1}{\sigma_2} \tag{1}$$

If x_1 and x_2 are estimated ignoring the correlation between them, effectively setting $\rho = 0$, we would therefore expect to observe a positive bias in $\hat{\beta}_2$ when ρ and $\hat{\beta}_1$ are both positive, because the second term in equation (1) is ignored. By symmetry, we would also expect to observe a positive bias in $\hat{\beta}_1$ for positive ρ and positive $\hat{\beta}_2$.

2 Additional simulation studies

In this section we present the results of further simulation studies investigating performance of the models for different levels of association between the longitudinal trajectories and the CVD event and for different levels of correlation between the usual level and the variability for Scenario 1.

Table 1 shows results for different values of the true logHR's α_0 and α_σ. All results are given for datasets with $n = 4$ measurements per individual. When $\alpha_\sigma = 0$, i.e. there is no association between the longitudinal variability and the time-to-event, the naive method has negative bias in

the usual level logHR due to regression dilution, but there is no bias in the variability logHR. By contrast, for methods LMM1 and JM1 there is slight positive bias in the variability logHR because the correlation between the usual level and the variability has been ignored. In this case, therefore, using the mixed effects model may lead to increased bias compared to the naive method because the model is misspecified. Results for larger values of the logHRs show substantial bias in both logHRs for the naive and LMM methods, with the naive method performing the worst. The joint models give little bias in the logHR for the usual level, but some negative bias for the variability logHR. Coverage probabilities are very much lower than the nominal 95% level, with those of the joint models being substantially closer than all other methods. These results suggest that for substantially larger effect sizes, or equivalently for substantially less variation in the usual levels or variabilities in the population, all methods require a greater number of measurements per individual to sufficiently reduce estimation bias.

Results for various values of the correlation parameter ρ are shown in Table 2. Again, each individual has $n = 4$ measurements. When there is negative correlation we find negative bias in both association parameters for the naive, LMM1 and JM1 methods, as we would expect from the arguments given in Section 1. Bias in the usual level logHR using the methods LMM1 and JM1, which ignore the correlation, increases with increasing ρ. But for the variability logHR the pattern is less clear because of the interplay between regression dilution bias and the bias incurred by ignoring correlation in the mixed effects model. For models LMM2 and JM2, which account for the correlation, we found minimal bias in estimated effects and coverage close to nominal values in all cases.

Table 1: Scenario 1 results with different levels of association between the usual level and the variability of the longitudinal outcome and the time-to-event. Presented are the true values, mean (standard deviation) of estimated log hazard ratios, root mean squared error and coverage over 1000 simulated datasets. Methods of analysis are (1) True values, where the true usual levels and variabilities are used as covariates, (2) Naive method, (3) LMM1, the two-stage approach with no correlation between the usual level and the variability, (4) LMM2, the two-stage approach with correlation, (5) JM1, the joint model with no correlation and (6) JM2, the joint model with correlation.

	**Usual level logHR α_0**				**Variability logHR α_σ**			
	True	Mean (SD)	RMSE	Coverage	True	Mean (SD)	RMSE	Coverage
**$\alpha_0 = 0.02$, $\alpha_\sigma = 0$**								
True values	0.02	0.0201 (0.0043)	0.0043	0.949	0	-0.0011 (0.0138)	0.0139	0.955
Naive	0.02	0.0175 (0.0038)	0.0045	0.893	0	0.0012 (0.0103)	0.0103	0.946
LMM1	0.02	0.0193 (0.0042)	0.0043	0.943	0	0.0062 (0.0165)	0.0176	0.935
LMM2	0.02	0.0199 (0.005)	0.005	0.944	0	-1e-04 (0.0192)	0.0192	0.953
JM1	0.02	0.0194 (0.0042)	0.0043	0.942	0	0.0043 (0.017)	0.0175	0.943
JM2	0.02	0.0201 (0.0051)	0.0051	0.944	0	-0.0024 (0.0198)	0.0199	0.956
**$\alpha_0 = 0.02$, $\alpha_\sigma = 0.05$**								
True values	0.02	0.02 (0.0043)	0.0043	0.94	0.05	0.0501 (0.0117)	0.0117	0.94
Naive	0.02	0.0208 (0.0037)	0.0038	0.947	0.05	0.0284 (0.0094)	0.0235	0.31
LMM1	0.02	0.0227 (0.0042)	0.005	0.892	0.05	0.0531 (0.0151)	0.0154	0.929
LMM2	0.02	0.0198 (0.0049)	0.0049	0.948	0.05	0.0511 (0.0175)	0.0175	0.931
JM1	0.02	0.023 (0.0042)	0.0052	0.883	0.05	0.0521 (0.0155)	0.0156	0.939
JM2	0.02	0.02 (0.0051)	0.0051	0.949	0.05	0.0502 (0.0183)	0.0183	0.936
**$\alpha_0 = 0.05$, $\alpha_\sigma = 0.02$**								
True values	0.05	0.0502 (0.0043)	0.0043	0.948	0.02	0.0201 (0.0112)	0.0112	0.942
Naive	0.05	0.044 (0.0037)	0.007	0.6	0.02	0.0125 (0.009)	0.0117	0.834
LMM1	0.05	0.0491 (0.0041)	0.0042	0.94	0.02	0.036 (0.0139)	0.0212	0.767
LMM2	0.05	0.0495 (0.0049)	0.0049	0.94	0.02	0.0216 (0.0161)	0.0161	0.944
JM1	0.05	0.0501 (0.0043)	0.0043	0.946	0.02	0.0339 (0.0145)	0.02	0.843
JM2	0.05	0.0505 (0.0051)	0.0051	0.945	0.02	0.0193 (0.0168)	0.0168	0.952
**$\alpha_0 = 0.1$, $\alpha_\sigma = 0.25$**								
True values	0.1	0.1002 (0.0043)	0.0043	0.94	0.25	0.251 (0.0106)	0.0106	0.952
Naive	0.1	0.082 (0.004)	0.0184	0.002	0.25	0.0972 (0.0097)	0.1531	0
LMM1	0.1	0.0911 (0.0043)	0.0099	0.386	0.25	0.1879 (0.0152)	0.0639	0.008
LMM2	0.1	0.0818 (0.0048)	0.0188	0.027	0.25	0.1759 (0.0183)	0.0763	0.007
JM1	0.1	0.1081 (0.0055)	0.0098	0.695	0.25	0.2191 (0.0191)	0.0363	0.589
JM2	0.1	0.0969 (0.0057)	0.0065	0.905	0.25	0.2096 (0.0215)	0.0458	0.503

Table 2: Scenario 1 results with different levels of correlation between the usual level and the variability of the longitudinal outcome. Presented are the true values, mean (standard deviation) of estimated log hazard ratios, root mean squared error and coverage over 1000 simulated datasets. Methods of analysis are (1) True values, where the true usual levels and variabilities are used as covariates, (2) Naive method, (3) LMM1, the two-stage approach with no correlation between the usual level and the variability, (4) LMM2, the two-stage approach with correlation, (5) JM1, the joint model with no correlation and (6) JM2, the joint model with correlation.

	**Usual level logHR α_0**				**Variability logHR α_σ**			
	True	Mean (SD)	RMSE	Coverage	True	Mean (SD)	RMSE	Coverage
$\rho = -0.5$								
True values	0.02	0.02 (0.0043)	0.0043	0.939	0.05	0.0497 (0.0127)	0.0127	0.957
Naive	0.02	0.0148 (0.0038)	0.0064	0.739	0.05	0.025 (0.01)	0.0269	0.255
LMM1	0.02	0.0165 (0.0042)	0.0055	0.883	0.05	0.0399 (0.0163)	0.0192	0.916
LMM2	0.02	0.0199 (0.0048)	0.0048	0.94	0.05	0.0507 (0.019)	0.019	0.951
JM1	0.02	0.0167 (0.0043)	0.0054	0.894	0.05	0.0382 (0.0164)	0.0202	0.898
JM2	0.02	0.0201 (0.005)	0.005	0.937	0.05	0.0493 (0.0192)	0.0192	0.955
$\rho = 0$								
True values	0.02	0.0198 (0.0038)	0.0038	0.946	0.05	0.0496 (0.0105)	0.0105	0.951
Naive	0.02	0.0177 (0.0036)	0.0042	0.892	0.05	0.0293 (0.0087)	0.0224	0.309
LMM1	0.02	0.0196 (0.0039)	0.004	0.945	0.05	0.0509 (0.0147)	0.0148	0.947
LMM2	0.02	0.0196 (0.0039)	0.004	0.946	0.05	0.0509 (0.0148)	0.0148	0.94
JM1	0.02	0.0199 (0.004)	0.004	0.943	0.05	0.0496 (0.015)	0.015	0.955
JM2	0.02	0.0199 (0.004)	0.004	0.941	0.05	0.0497 (0.015)	0.015	0.949
$\rho = 0.2$								
True values	0.02	0.0201 (0.0037)	0.0037	0.95	0.05	0.0501 (0.0105)	0.0105	0.959
Naive	0.02	0.0191 (0.0035)	0.0036	0.949	0.05	0.0294 (0.0088)	0.0224	0.32
LMM1	0.02	0.021 (0.0039)	0.004	0.949	0.05	0.0525 (0.0147)	0.0149	0.935
LMM2	0.02	0.02 (0.004)	0.004	0.954	0.05	0.0509 (0.0151)	0.0151	0.939
JM1	0.02	0.0213 (0.0039)	0.0042	0.945	0.05	0.0514 (0.0151)	0.0152	0.94
JM2	0.02	0.0202 (0.004)	0.004	0.956	0.05	0.0497 (0.0154)	0.0154	0.949
$\rho = 0.5$								
True values	0.02	0.02 (0.0043)	0.0043	0.94	0.05	0.0501 (0.0117)	0.0117	0.94
Naive	0.02	0.0208 (0.0037)	0.0038	0.947	0.05	0.0284 (0.0094)	0.0235	0.31
LMM1	0.02	0.0227 (0.0042)	0.005	0.892	0.05	0.0531 (0.0151)	0.0154	0.929
LMM2	0.02	0.0198 (0.0049)	0.0049	0.948	0.05	0.0511 (0.0175)	0.0175	0.931
JM1	0.02	0.023 (0.0042)	0.0052	0.883	0.05	0.0521 (0.0155)	0.0156	0.939
JM2	0.02	0.02 (0.0051)	0.0051	0.949	0.05	0.0502 (0.0183)	0.0183	0.936
$\rho = 0.8$								
True values	0.02	0.0201 (0.0055)	0.0055	0.959	0.05	0.0503 (0.015)	0.015	0.955
Naive	0.02	0.0237 (0.0041)	0.0055	0.831	0.05	0.0242 (0.0098)	0.0276	0.217
LMM1	0.02	0.0256 (0.0046)	0.0073	0.767	0.05	0.0495 (0.015)	0.015	0.948
LMM2	0.02	0.0202 (0.0077)	0.0077	0.951	0.05	0.0506 (0.0245)	0.0245	0.946
JM1	0.02	0.0259 (0.0047)	0.0075	0.749	0.05	0.0485 (0.0154)	0.0155	0.953
JM2	0.02	0.02 (0.008)	0.008	0.949	0.05	0.051 (0.026)	0.026	0.946

3 ARIC study baseline characteristics

Table 3 shows baseline characteristics of individuals in the reduced and full ARIC datasets at the study baseline. The age and cholesterol levels are similar between the two datasets, but the reduced dataset has a lower proportion of males, smokers and diabetics than the full dataset.

Table 3: Baseline characteristics of individuals in the reduced and full ARIC datasets; mean (SD) unless stated otherwise.

	Reduced dataset n = 10,019	Full dataset n = 13,161
Age, years	54.1 (5.7)	54.4 (5.7)
Sex (male), n (%)	4286 (42.8)	5924 (45.0)
Total cholesterol, mmol/L	5.5 (1.0)	5.5 (1.1)
HDL cholesterol, mmol/L	1.4 (0.4)	1.4 (0.4)
Smoker, n(%)	2372 (23.7)	3671 (27.9)
Diabetic, n(%)	817 (8.2)	1407 (10.7)

4 JAGS code

The following JAGS code fits the linear mixed effects model LMM1 without correlation between the SBP usual level and SBP variability, equations (2) and (4) in the main text. The data takes the following format: `y` is a vector containing all SBP measurements from all individuals and `subject` is a vector identifying which individual the measurement comes from. In the code `BP` is a vector of the true SBP usual levels and `BPSD` is a vector of the true SBP residual SDs.

```
model{
# Normally distributed repeated measurements
    for(j in 1:Nobs) {
        y[j] ~ dnorm(BP[subject[j]], tau.BPSD[subject[j]])
    }
# Random effects distribution
    for(i in 1:N){
        BP[i] ~ dnorm(mu.BP, tau.BP)

        tau.BPSD[i] <- pow(BPSD[i], -2)
        BPSD[i] <- exp(log.BPSD[i])
        log.BPSD[i] ~ dnorm(mu.log.BPSD, tau.log.BPSD)
    }
# Priors
    mu.BP ~ dnorm(0,1.0E-4)
    tau.BP <- pow(sd.BP, -2)
    sd.BP ~ dunif(0, 100)
    mu.log.BPSD ~ dnorm(0,1.0E-4)
    tau.log.BPSD <- pow(sd.log.BPSD, -2)
    sd.log.BPSD ~ dunif(0, 100)
}
```

The following JAGS code fits the joint model JM1 for the ARIC data without correlation between

the SBP usual level and SBP variability. The repeated measurement data is as described above and survival data consists of a dataset in which each individual has multiple rows, one for each piecewise-constant time interval: `start` is the start of the time interval, `end` is the end of the time interval or event time, `survid` specifies the individual, `period` specifies the time interval and `event` is an indicator, equal to 0 if no event occurred and equal to 1 if an event occurred at the end time. In addition the vectors `tchol`, `hdl`, `age`, `smoke`, `diab` and `sex` contain baseline covariates.

```
# For the ones trick
data{
    for(i in 1:nrow){
        ones[i]<-1
    }
}
model{
# Normally distributed repeated measurements
    for(j in 1:Nobs){
        y[j]~dnorm(BP[id[j]],tau.BPSD[id[j]])
    }

# Survival model
    for(i in 1:nrow){
# Hazard for time interval i (constant)
        h[i]<-exp(eta[period[i]]+alpha1*BP[survid[i]]+alpha2*BPSD[survid[i]]
              +gamma[1]*tchol[i]+gamma[2]*hdl[i]+gamma[3]*age[i]+gamma[4]*smoke[i]
              +gamma[5]*diab[i]+gamma[6]*sex[i])
# Integrated hazard for individual i from start time to end time of at risk period
        H[i]<-h[i]*(end[i]-start[i])
# Survival function
        S[i]<-exp(-H[i])
# Density function of survival time
        f[i]<-h[i]*S[i]
# Likelihood for survival data using the ones trick
        ones[i]~dbern(p[i])
        p[i]<-L[i]
# Likelihood contribution for period period[i], individual id[i]
        L[i]<-pow(f[i],event[i])*pow(S[i],1-event[i])
    }

# Random-effects distribution
    for(i in 1:N){
        BP[i] ~ dnorm(mu.BP, tau.BP)

        tau.BPSD[i] <- pow(BPSD[i], -2)
        BPSD[i] <- exp(log.BPSD[i])
        log.BPSD[i] ~ dnorm(mu.log.BPSD, tau.log.BPSD)
    }

# Priors
    mu.BP ~ dnorm(0,1.0E-4)
    tau.BP <- pow(sd.BP, -2)
    sd.BP ~ dunif(0, 100)
    for(k in 1:nk){
        eta[k]~dnorm(0,1.0E-4)
    }
    alpha1~dnorm(0,1.0E-4)
    alpha2~dnorm(0,1.0E-4)
    for(k in 1:6){
        gamma[k]~dnorm(0,1.0E-4)
    }
```

```
    mu.log.BPSD ~ dnorm(0,1.0E-4)
    tau.log.BPSD <- pow(sd.log.BPSD, -2)
    sd.log.BPSD ~ dunif(0, 100)
}
```

The following code fits the linear mixed model LMM2, which includes the correlation between the SBP usual level and the SBP variability. The code to fit joint model JM2 can be adapted from the code for model JM1 in a similar way.

```
model{
# Normally distributed repeated measurements
    for(j in 1:Nobs) {
        y[j] ~ dnorm(BP[subject[j]], tau.BPSD[subject[j]])
    }
# Random-effects distribution
    for(i in 1:N){
        BP[i] ~ dnorm(mu.BP, tau.BP)

        tau.BPSD[i] <- pow(BPSD[i], -2)
        BPSD[i] <- exp(log.BPSD[i])

# Conditional normal distribution for logBPSD[i], conditional on BP[i]
        log.BPSD[i] ~ dnorm(mu.log.BPSD.knowing.BP[i],  sd.log.BPSD.knowing.BP)
        mu.log.BPSD.knowing.BP[i] <- mu.log.BPSD + sd.log.BPSD/sd.BP*rho*(BP[i] - mu.BP)
    }

# Priors
    mu.BP ~ dnorm(0,1.0E-4)
    tau.BP <- pow(sd.BP, -2)
    sd.BP ~ dunif(0, 100)
    mu.log.BPSD ~ dnorm(0,1.0E-4)
    tau.log.BPSD <- pow(sd.log.BPSD, -2)
    sd.log.BPSD ~ dunif(0, 100)
    sd.log.BPSD.knowing.BP <- tau.log.BPSD*1/(1-pow(rho,2))
    rho ~ dunif(-1,1)
}
```

The following code fits the linear mixed model LMM1, adjusting the longitudinal model for baseline risk factors. Models LMM2, JM1 and JM2 can be adapted in a similar way. Here `covariates` is a matrix of baseline covariates.

```
model{
# Normally distributed repeated measurements, adjusted for baseline CVD risk factors
    for(j in 1:Nobs) {
        y[j] ~ dnorm(mu.y[j], tau.BPSD[subject[j]])
        mu.y[j] <- BP[subject[j]] + inprod(covariates[subject[j],1:6],beta[1:6])
    }
# Random-effects distribution
    for(i in 1:N){
        BP[i] ~ dnorm(mu.BP, tau.BP)

        tau.BPSD[i] <- pow(BPSD[i], -2)
            BPSD[i] <- exp(log.BPSD[i])
            log.BPSD[i] ~ dnorm(mu.log.BPSD, tau.log.BPSD)
    }
# Priors
    mu.BP ~ dnorm(0,1.0E-4)
```

```
    tau.BP <- pow(sd.BP, -2)
    sd.BP ~ dunif(0, 100)
    mu.log.BPSD ~ dnorm(0,1.0E-4)
    tau.log.BPSD <- pow(sd.log.BPSD, -2)
    sd.log.BPSD ~ dunif(0, 100)
    for(k in 1:6){
        beta[k] ~ dnorm(0,1.0E-4)
    }
}
```

The following code fits the random intercept and slope linear mixed model LMM3 with no correlation between the random effects and the SBP variability. Model JM3 can be adapted from model JM1 in a similar way.

```
model{
# Normally distributed repeated measurements with random intercept and random slope
    for(j in 1:Nobs) {
        y[j] ~ dnorm(mu.y[j], tau.BPSD[subject[j]])
        mu.y[j] <- BP[subject[j]] + BPslope[subject[j]]*time[j]
    }
# Random-effects distribution
    for(i in 1:N){
        BP[i] ~ dnorm(mu.BP, tau.BP)

# Conditional normal distribution for BPslope[i], conditional on BP[i]
        BPslope[i] ~ dnorm(mu.BPslope.knowing.BP[i],  tau.BPslope.knowing.BP)
        mu.BPslope.knowing.BP[i] <- mu.BPslope+sd.BPslope/sd.BP*rho.BP.BPslope*(BP[i]
         -mu.BP)

        tau.BPSD[i] <- pow(BPSD[i], -2)
        BPSD[i] <- exp(log.BPSD[i])
        log.BPSD[i] ~ dnorm(mu.log.BPSD, tau.log.BPSD)
    }

# Priors
    mu.BP ~ dnorm(0,1.0E-4)
    tau.BP <- pow(sd.BP, -2)
    sd.BP ~ dunif(0, 100)
    mu.BPslope ~ dnorm(0,1.0E-4)
    tau.BPslope <- pow(sd.BPslope, -2)
    sd.BPslope ~ dunif(0, 100)
    tau.BPslope.knowing.BP <- tau.BPslope*1/(1-pow(rho.BP.BPslope,2))
    rho.BP.BPslope ~ dunif(-1,1)
    mu.log.BPSD ~ dnorm(0,1.0E-4)
    tau.log.BPSD <- pow(sd.log.BPSD, -2)
    sd.log.BPSD ~ dunif(0, 100)
}
```

The following code fits the linear mixed model LMM4 which includes random intercepts and slopes and correlations between all random effects and SBP variability. Model JM3 can be adapted from model JM1 in a similar way.

```
model{
# Normally distributed repeated measurements with random intercept and random slope
    for(j in 1:Nobs) {
        y[j] ~ dnorm(mu.y[j], tau.BPSD[subject[j]])
        mu.y[j] <- BP[subject[j]] + BPslope[subject[j]]*time[j]
```

```
    }
# Random-effects distribution
    for(i in 1:N){
        BP[i] ~ dnorm(mu.BP, tau.BP)

# Conditional normal distribution for BPslope[i], conditional on BP[i]
        BPslope[i] ~ dnorm(mu.BPslope.knowing.BP[i],  tau.BPslope.knowing.BP)
        mu.BPslope.knowing.BP[i] <- mu.BPslope+sd.BPslope/sd.BP*rho.BP.BPslope*(BP[i]
        -mu.BP)

# Conditional normal distribution for logBPSD[i], conditional on BP[i] and BPslope[i]
        log.BPSD[i] ~ dnorm(mu.logBPSD.knowing.BP.BPslope[i],
                                 tau.logBPSD.knowing.BP.BPslope)
        mu.logBPSD.knowing.BP.BPslope[i] <- mu.log.BPSD+sd.log.BPSD/sd.BP*(rho.BP.logBPSD
        -rho.BP.BPslope*rho.BPslope.logBPSD)/(1-pow(rho.BP.BPslope,2))*(BP[i] - mu.BP)
        +sd.log.BPSD/sd.BPslope*(rho.BPslope.logBPSD-rho.BP.BPslope*rho.BP.logBPSD)
        /(1-pow(rho.BP.BPslope,2))*(BPslope[i] - mu.BPslope)
        tau.BPSD[i] <- pow(BPSD[i], -2)
        BPSD[i] <- exp(log.BPSD[i])
    }

# Priors
    mu.BP ~ dnorm(0,1.0E-4)
    tau.BP <- pow(sd.BP, -2)
    sd.BP ~ dunif(0, 100)
    mu.BPslope ~ dnorm(0,1.0E-4)
    tau.BPslope <- pow(sd.BPslope, -2)
    sd.BPslope ~ dunif(0, 100)
    tau.BPslope.knowing.BP <- tau.BPslope*1/(1-pow(rho.BP.BPslope,2))
    rho.BP.BPslope ~ dunif(-1,1)
    rho.BP.logBPSD ~ dunif(-1,1)
    rho.BPslope.logBPSD ~ dunif(-1,1)
    mu.log.BPSD ~ dnorm(0,1.0E-4)
    tau.log.BPSD <- pow(sd.log.BPSD, -2)
    sd.log.BPSD ~ dunif(0, 100)
    var.logBPSD.knowing.BP.BPslope <- pow(sd.log.BPSD,2)*(1-(pow(rho.BP.logBPSD,2)
    +pow(rho.BPslope.logBPSD,2)-2*rho.BP.BPslope*rho.BP.logBPSD*rho.BPslope.logBPSD)
    /(1-pow(rho.BP.BPslope,2)))
    tau.logBPSD.knowing.BP.BPslope <- pow(var.logBPSD.knowing.BP.BPslope,-1)
}
```
